# Incremental prognostic value of triglyceride glucose index additional to coronary artery calcium score in asymptomatic low-risk population

**DOI:** 10.1186/s12933-022-01620-7

**Published:** 2022-09-23

**Authors:** Shinjeong Song, Su‑Yeon Choi, Hyo Eun Park, Hae‑Won Han, Sung Hak Park, Jidong Sung, Hae Ok Jung, Ji Min Sung, Hyuk‑Jae Chang

**Affiliations:** 1 Division of Cardiology, Department of Internal Medicine, Ewha Womans University Hospital, Ewha Womans University College of Medicine, Seoul, South Korea; 2 Yonsei University College of Medicine, Yonsei University Health System, Seoul, Korea; 3 Division of Cardiology, Healthcare System Gangnam Center, Seoul National University Hospital, Seoul, South Korea; 4 Department of Internal Medicine, Gangnam Heartscan Clinic, Seoul, South Korea; 5 Division of Radiology, Gangnam Heartscan Clinic, Seoul, South Korea; 6 Division of Cardiology, Heart Stroke & Vascular Institute, Samsung Medical Center, Seoul, South Korea; 7 Division of Cardiology, Seoul St. Mary’s Hospital, College of Medicine, The Catholic University of Korea, Seoul, South Korea; 8 Division of Cardiology, Severance Cardiovascular Hospital, Yonsei University College of Medicine, Yonsei University Health System. 50‑1 Yonsei‑ro, Seodaemun‑gu, 03722 Seoul, South Korea

**Keywords:** Triglyceride glucose index, Coronary artery calcification, Atherosclerotic cardiovascular disease

## Abstract

**Background:**

The triglyceride glucose (TyG) index has been suggested as a reliable surrogate marker of insulin resistance which is a substantial risk factor for atherosclerotic cardiovascular disease (ASCVD). Several recent studies have shown the relationship between the TyG index and cardiovascular disease; however, the role of the TyG index in coronary artery calcification (CAC) progression has not been extensively assessed especially in low-risk population.

**Methods:**

We enrolled 5775 Korean adults who had at least two CAC evaluations. We determined the TyG index using ln (fasting triglycerides [mg/dL] x fasting glucose [mg/dL]/2). The CAC progression was defined as either incident CAC in a CAC-free population at baseline or an increase of ≥ 2.5 units between the square roots of the baseline and follow-up coronary artery calcium scores (CACSs) of subjects with detectable CAC at baseline.

**Results:**

CAC progression was seen in 1,382 subjects (23.9%) during mean 3.5 years follow-up. Based on the TyG index, subjects were stratified into four groups. Follow-up CACS and incidence of CAC progression were markedly elevated with rising TyG index quartiles (group I [lowest]:17.6% vs. group II:22.2% vs. group III:24.6% vs. group IV [highest]: 31.3%, p < 0.001). In multivariate logistic regression analysis, the TyG index was independent predictor of CAC progression (odds ratio: 1.57; 95% confidence interval: 1.33 to 1.81; p < 0.001) especially in baseline CACS ≤ 100 group.

**Conclusion:**

The TyG index is an independent predictor of CAC progression in low-risk population. It adds incremental risk stratification over established factors including baseline CACS.

## Introduction

The progression of cardiovascular (CV) disease associated with atherosclerosis is strongly associated with the risk of cardiovascular morbidity and mortality. Coronary artery calcification (CAC) is an effective marker for atherosclerosis cardiovascular disease (ASCVD) and predicts adverse outcomes. Therefore, CV risk is evaluated by the coronary artery calcium score (CACS), which is typically determined by computed tomography. In addition to baseline CACS and traditional CV risk factors, CAC progression is known as a strong predictor of mortality [1–3].

The triglyceride glucose (TyG) index has been proposed as a reliable surrogate marker for insulin resistance (IR), a practical risk factor for ASCVD [4–6]. In addition, several studies have identified a strong relationship between the TyG index and atherosclerosis under various clinical conditions [7, 8] and relatively high CV risk patients [9–11].

It is also well known that the benefits of statin therapy are also seen in low-risk individuals. The 2019 ACC/AHA Primary Prevention Guidelines recommend that people within the of 10-years ASCVD borderline risk range (5-7.5%) consider using statins in consideration of cost efficiency and various clinical situations [12]. However, in relatively low-risk adults with atherosclerosis cardiovascular disease (ASCVD), data on the association of changes in the TyG index and CAC are limited.

Therefore, in the present study, we evaluated the association of TyG in predicting CACS progression, which is known to be well related to ASCVD, in a low-risk patient group.

## Methods

### Study population and design

**Fig. 1:**
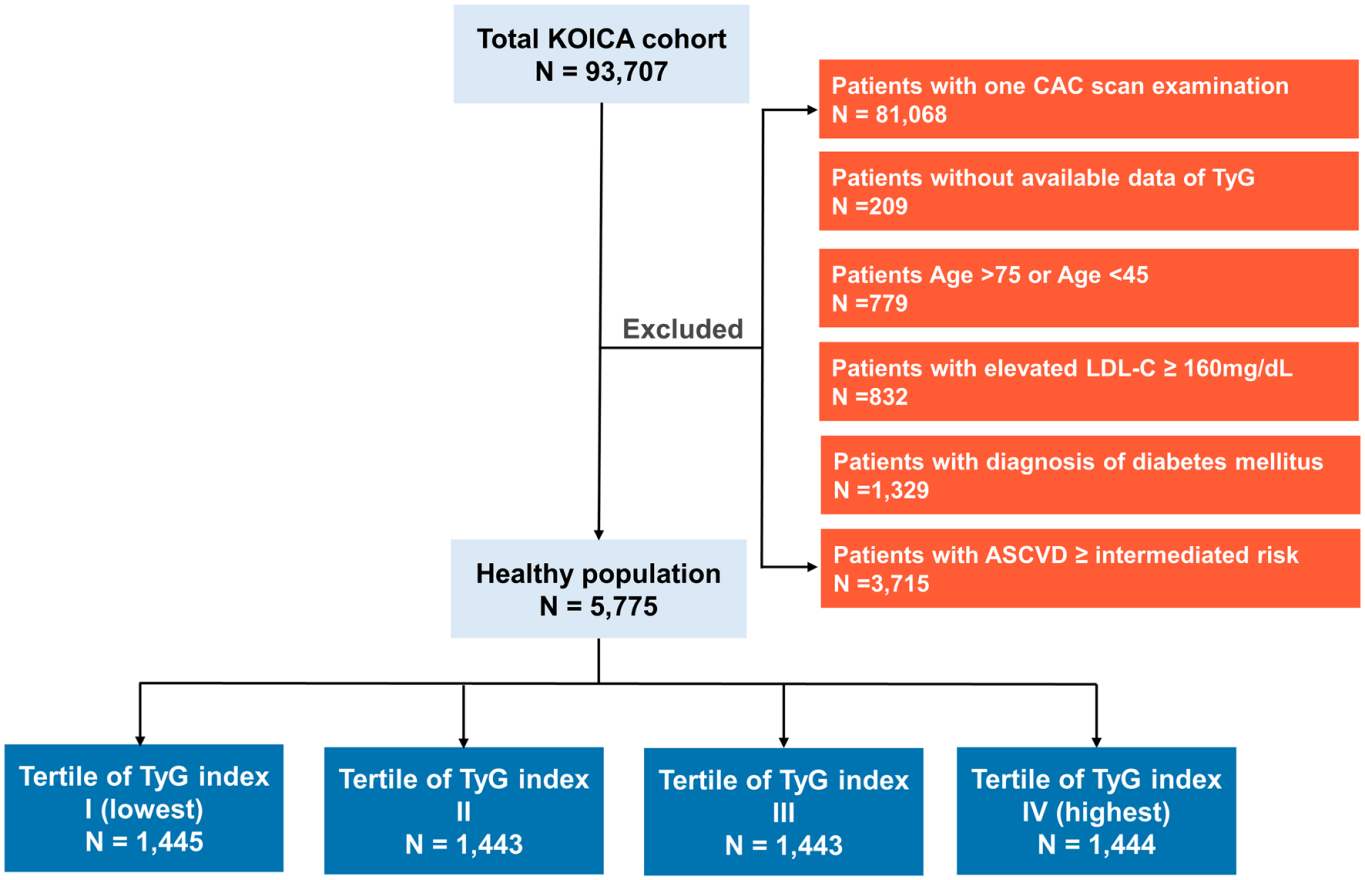
Population flow chart

Data from the Korea Initiatives on Coronary Artery Calcification (KOICA) registry were analyzed in the present study. The KOICA is a retrospective, multicenter, and observational registry with single ethnicity in the setting of self-referral for asymptomatic subjects who underwent general health examination at six healthcare centers in South Korea [13]. Overall, 93,707 subjects were enrolled in KOICA registry from December 2012 to April 2017. Self-reported medical questionnaires were used to obtain information on previous medical history. All data were obtained during visits to each healthcare center. Among the 93,707 subjects from this registry, subjects with only on coronary artery calcium scan examination (n = 81,068), patients without available data TyG (n = 209), people under the age of 45 or over 75 (n = 779), or LDL-C cholesterol ≥ 160 mg/dL (n = 832), or diagnosis of diabetes mellitus (n = 1329), or atherosclerosis cardiovascular disease risk over low (n = 3715) were excluded. To exclude diabetic patients, we excluded both those who answered that they were currently taking antidiabetic medications in a patient self-questionnaire and those who met diagnostic criteria for diabetes from testing at the time of enrollment. Finally, 5775 subjects were analyzed (Fig. 1).

**Fig. 2:**
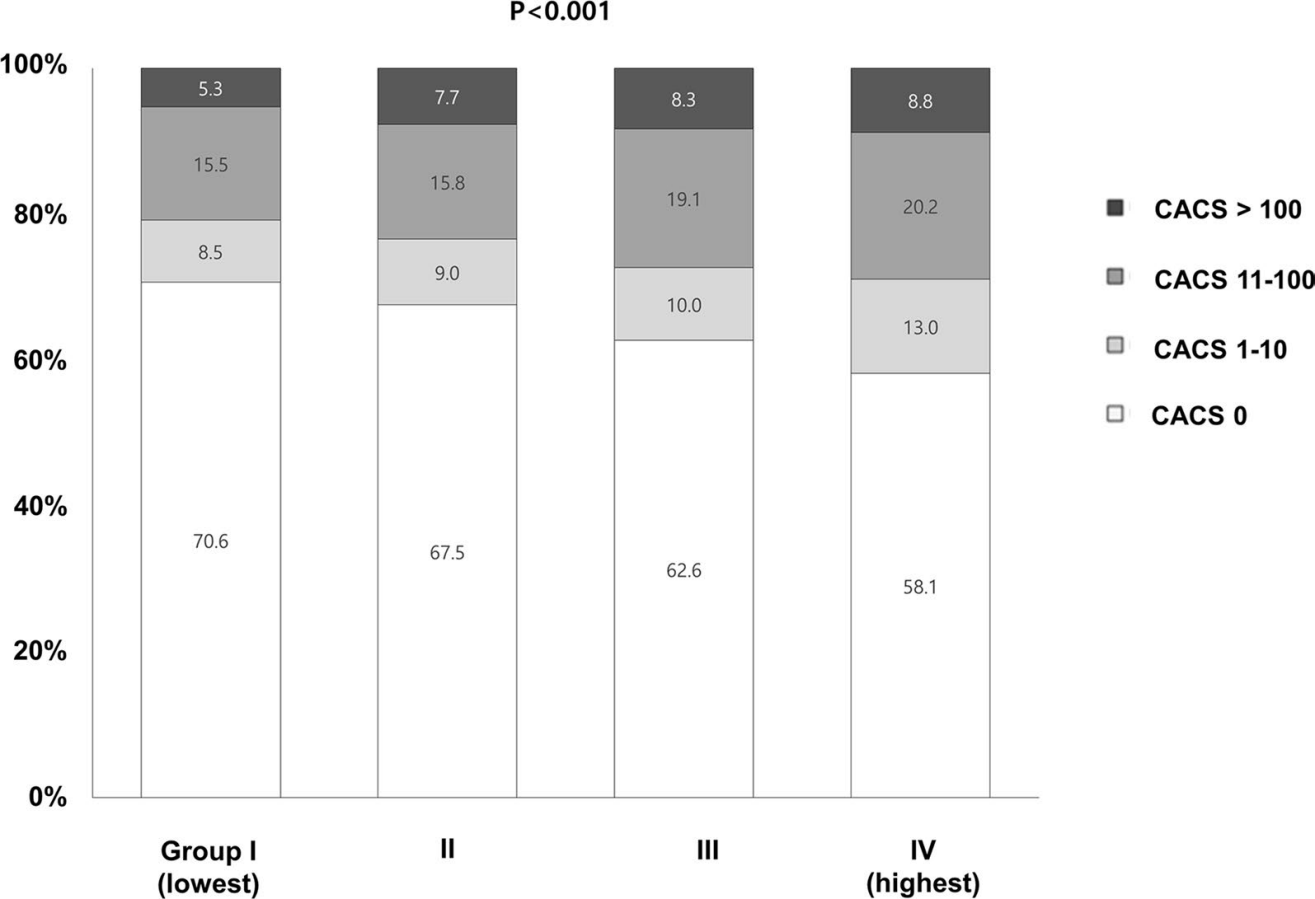
Comparison of baseline coronary artery calcification according to triglyceride glucose index tertiles. * CACS* coronary artery calcium score, *GroupI* lowest TyG index group, *Group IV* highest TyG index group

**Fig. 3:**
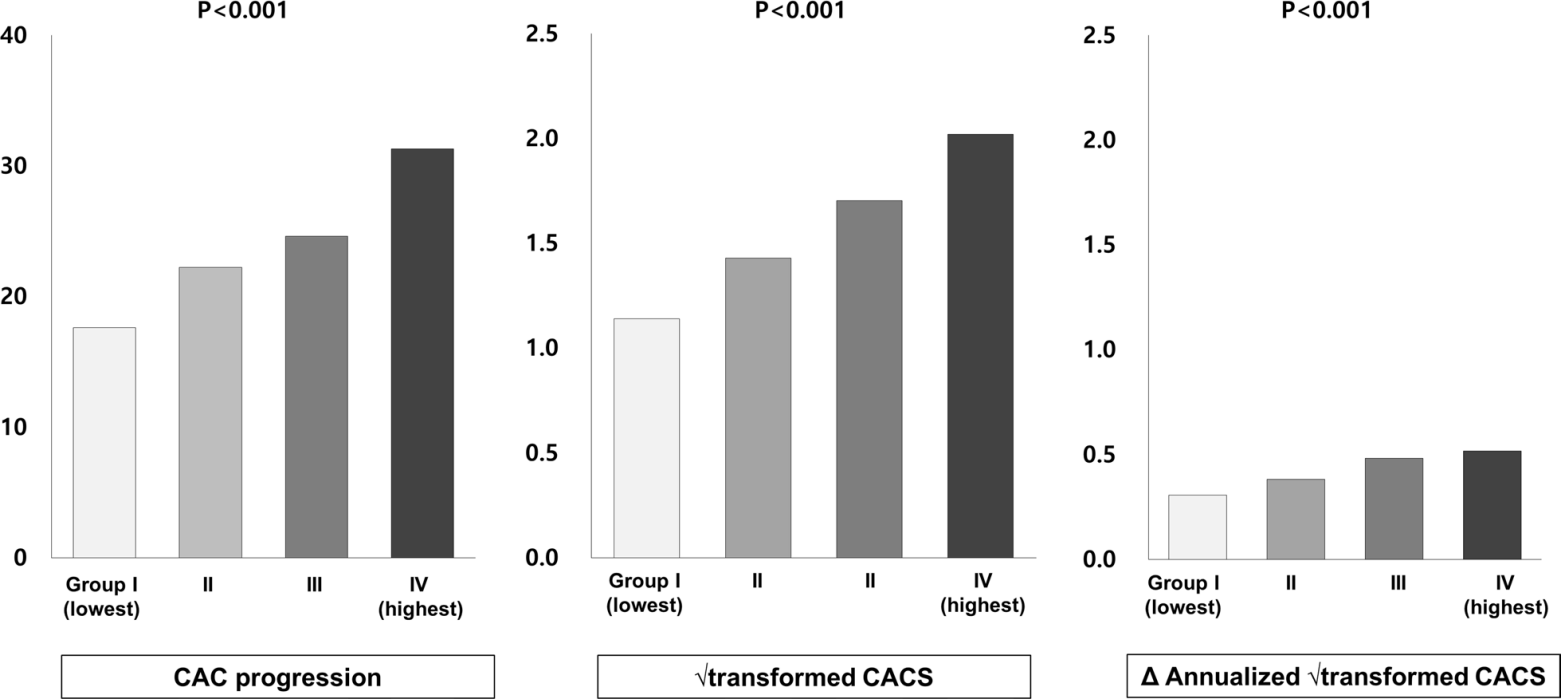
Change of CAC according TyG index tertiles. * CAC* coronary artery calcium, *TyG* Triglyceride glucose

**Fig. 4:**
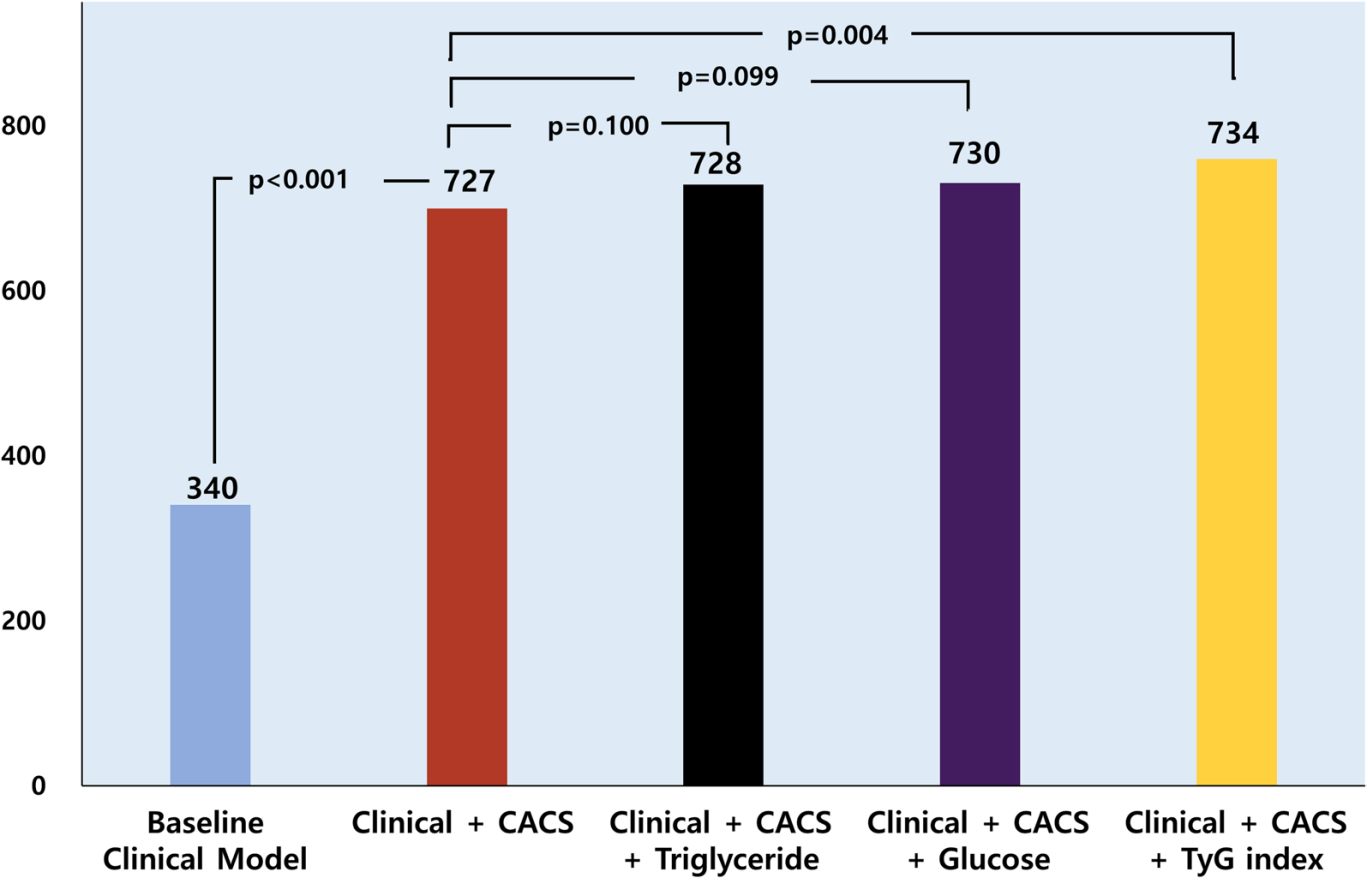
Incremental value of TyG index to predict CAC progression. * TyG* Triglyceride glucose, *CAC* coronary artery calcium

**Fig. 5:**
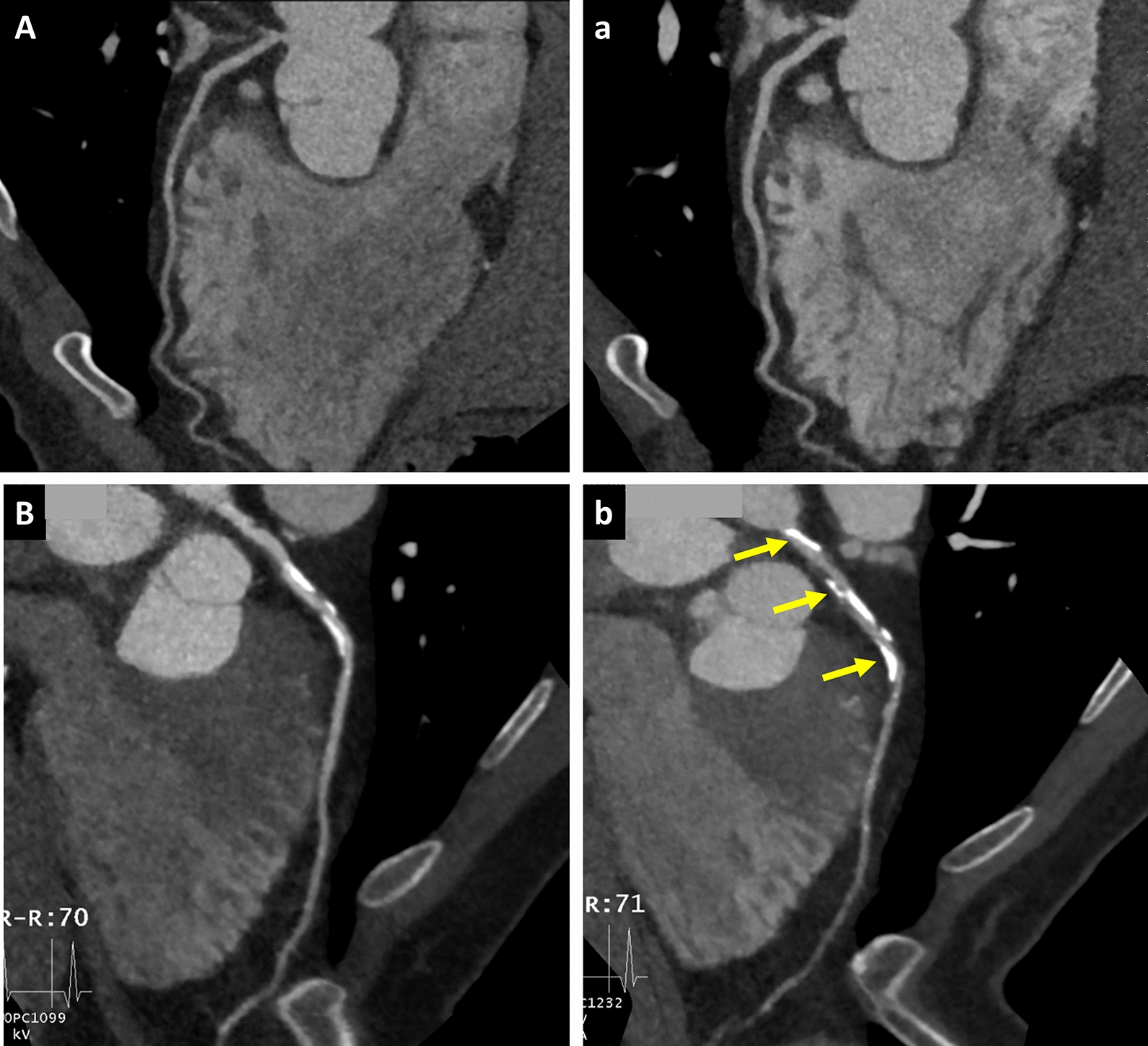
Two consecutive coronary CT angiography studies showing CAC and its correlation with TyG index. Automatically generated curved multiplanar reformation of image data left anterior descending coronary artery in an asymptomatic nondiabetic 59-year-old man performed in December 2012 (**A**) and in May 2015 (a), and asymptomatic nondiabetic 56-year-old man performed in January 2012 (**B**) and in June 2014 (b). Patient with low CACS and low TyG index group did not develop coronary artery calcification (**A**, a). But there is progression of overall calcification volume as well as dense calcification in patient classified as low-risk patient but with high CACS and high TyG index group at baseline (**B**, b). * CACS* coronary artery calcium score, *TyG* Triglyceride glucose

### Data collection

Information on hypertension, diabetes, hyperlipidemia, and smoking was systematically collected for each subject. Weight, height, and blood pressure were measured during the healthcare center visits. Weight and height were measured during the subjects wore light clothing without shoes. The body mass index was calculated as weight (kg)/height (m^2^). Blood pressure of the right arm was measured using an automatic manometer after resting for at least more than 5 min. All blood samples including total cholesterol, triglyceride, high-density lipoprotein cholesterol (HDL-C) low-density lipoprotein cholesterol (LDL-C), creatinine, glucose, and glycated hemoglobin A1C (HbA1C) levels were obtained after at least 8 h of fasting and analyzed. All methods were performed in accordance with the relevant guidelines and regulations.

### Definitions

The TyG index was determined using ln (triglycerides [mg/dL] ×glucose [mg/dL]/2). All subjects were categorized into four groups based on the quartiles of the TyG index level. CAC scores were calculated using the Agatston method [14]. CAC progression was defined as a diference ≥ 2.5 between the square roots (√) of the baseline and follow-up CACSs (Δ√transformed CACS) considering inter-scan variability [15]. Annualized Δ√transformed CACS was defined as Δ√transformed CACS divided by the inter-scan period.

Multi-detector CT scanners used to assess CAC had at least 16 slices (Philips Brilliance 256 iCT, and Philips Brilliance 40-channel multi-detector CT, GE 64-slice Lightspeed, Siemens 16-slice Sensation). All centers performed standard prospective or retrospective methods.

Diabetes was defined as either a fasting glucose level ≥ 126 mg/dL, HbA1C level ≥ 6.5%, a referral diagnosis of diabetes, or use of anti-diabetic treatment [16].

### Statistical analysis

Continuous variables are expressed as the mean ± standard deviation; Categorical variables are presented as proportions. After checking the distribution status of variables, the one-way analysis of variance test was used to analyze continuous variables and the χ2 or Fisher’s exact test was used to analyze categorical variables. Multivariate linear regression analysis was used for the association of clinical variables with progression of CACS and annualized Δ√transformed CACS in overall participants. Multivariate logistic regression analysis was performed to identify independent predictors for CAC progression according to categorical baseline CACS. Sequential Cox models were performed to determine the incremental prognostic benefit of CACS over clinical data, triglyceride over both, and then glucose over the rest and in TyG index (combination parameter combing TG and glucose). A statistically significant increase in the global chi-square test of the model defined incremental prognostic value. All statistical analyses were performed using the Statistical Package for the Social Sciences version 19 (SPSS, Chicago, Illinois). A P-value < 0.05 was considered significant in all analyses.

### Ethical statement

The appropriate institutional review board committees of each center approved the protocol of present study.

## Results

### Baseline characteristics

The mean age of the 5,775 subjects at baseline was 49 ± 5 years, and 82.6% of subjects were men (n = 4,771). The included subjects were stratified into four groups based on TyG index level. The biochemical parameters and clinical characteristics of the study subjects are presented in Table 1. TC, LDL-C, FPG, and TG levels and BMI, DBP, and SBP were elevated, whereas HDL-C Śwas decreased in subjects with a high TyG index. Baseline CACS values were also higher with a higher TyG index (Fig. 2).

### CACS change according to TyG index level

Table 1 presents the follow-up CACS according to TyG index. The average follow-up period was 3.5 ± 1.9 years. Follow-up CACS and incidence of CAC progression increased significantly with a rise in TyG index quartiles. Figure 3 shows that both the Δ√transformed CACS (Group I[GI] and annualized Δ√transformed CACS increased significantly with a rise in TyG index quartiles. The group with a highest TyG index had greater Δ√transformed CACS and annualized Δ√transformed CACS.

### TyG index and the risk of CAC progression according to the baseline CACS

Regarding the relationship between TyG index and CAC progression according to baseline CACS, the TyG index (per unit increase) was associated with an increased risk of CAC progression in baseline CACS group 0, 1–10 and 10–100 after adjusting for age, sex, BMI, systolic blood pressure (SBP), diastolic blood pressure (DBP) HDL-C, LDL-C, current smoking, 10-year ASCVD risk, and serum creatinine level. However, this association of TyG index with CAC progression was not observed in the group with baseline CACS > 100 (Table 2).

### Incremental prognostic value of TyG in CACS progression prediction

Significant increases in global chi-square test for the Cox proportional hazards models occurred after the addition of baseline CACS to the baseline clinical model (p < 0.05) demonstrating the incremental prognostic utility of CACS in progression prediction. Additionally, the inclusion of TyG in the model led to further incremental improvement in predictive accuracy above the overall combined model (including baseline clinical and CACS) (Figs. 4 and 5).

## Discussion

In the current study, we demonstrated a significant association between CAC progression and TyG index in a group with a low to borderline 10-years ASCVD risk. Even after adjusting for cardiovascular risk factors, there were an independent association and incremental prognostic value of TyG index with CAC progression. These results are consistent with previous studies which showed association between increased TyG index level and traditional CVD risk factors [7, 17–19].

Although the mechanism underlying the association between TyG and CAC is still unclear, the TyG index is a surrogate marker of IR, which may be important. In clinical practice, TG and glycemia are among the classic markers of cardiometabolic risk. Alteration in the levels of these markers is directly associated with IR, progression of atherosclerosis and genesis of CVD. IR is defined as a clinical or experimental condition in which glucose absorption and use are impaired [20]. Previous studies have shown that IR induces an imbalance in glucose and lipid metabolism, which is associated with cardiovascular risk in various population groups [20–22]. HOMA-IR is a comparatively extensive method for IR assessment [23]. The TyG index is strongly correlated with HOMA-IR and hyperinsulinemia-normal blood glucose clamp (HIEC) and even outperforms HOMA-IR [5, 24].

In recent years, many clinical studies have shown that the TyG index correlates with the risk of developing cardiovascular disease. Sánchez Iñigo et al. and Li et al. showed that healthy participants with elevated TyG index are at higher risk of cardiovascular events in each retrospective cohort studies [18, 25]. Thai et al. reported that elevated TyG index is correlated with the incidence and severity of coronary stenosis in patients with DM [26]. Park et al. reported that the TyG index is an independent predictor of the coronary artery calcification progression [27]. Liu et al. also reported that the high TyG index increases the risk of asymptomatic myocardial damage [28]. Various studies have shown that patients with high TyG index are more likely to develop hypertension and diabetes, [19, 29] which was also confirmed in the present research. In addition, several studies proposed that the TyG index can be also prognostic factor of major adverse cardiac events in patients with acute coronary syndrome and/or percutaneous coronary intervention (PCI) and DM [30–32]. Based on these studies, some suggested using the TyG index as an indicator for determining the secondary prevention policy [33].

In several previous studies, it is well known that TyG index is a prognostic factor related to cardiovascular risk in several situations, but its meaning is relatively unknown in asymptomatic and low risk patients. The use of the CAC score in asymptomatic subjects at intermediate risk, as determined by traditional clinical stratification methods, such as the Framingham risk score, is considered appropriate/recommended with a class IIb level of evidence by 2019 ACC/AHA Guideline on the Primary Prevention of Cardiovascular Disease. The use of the CAC score is not indicated in high-risk patients, because aggressive preventive measures would already be indicated in such patients. Within the group of patients classified as being at low risk, we have attempted to identify a subgroup with a significant long-term risk of a cardiovascular event, for which preventive measures should be adopted [12].

In this study, we demonstrate that baseline CACS is also a meaningful factor for CACS progression to predict cardiovascular events in the low to borderline risk group, and the TyG index is also an independent factor for CACS progression in this group. We also found that TyG index is an incremental value that predicts CACS progression in addition to the clinical factor and baseline CAC. Therefore, considering the rationale for the prospective factor of the cardiovascular disorder of TyG in several previous studies, it is expected that it will be beneficial to consider both CACS and TyG in addition to the existing well-known clinical factor when determining the use of statins for primary prevention in a group below borderline risk.

## Limitation

There are some limitations to the present study. The present study only included subjects who experienced at least two CAC scan examinations with available data on the TyG index and diabetic status from the KOICA registry. So, potential selection bias might be present. Second, this study is a retrospective observational study, we tried to exclude both those who answered that they were currently taking antidiabetic medications in a patient self-questionnaire and those who met diagnostic criteria for diabetes from testing at the time of enrollment. But as this is a retrospective study, it is influenced by medications containing lipid-lowering agents or glucose lowering drugs used before enrollment. Third, we only evaluated the association between the baseline TyG index and CAC progression. Fourth, the homeostatic model assessment of IR was not analyzed because insulin levels were not measured in the KOICA registry. Finally, the present study included only the Korean population.

## Conclusion

This study is unique in that we identified the incremental value of the TyG index for CAC progression in addition to baseline CAC status in a large sample of low to borderline asymptomatic risk adults.

**Table 1:** Clinical characteristics of the study cohort

	Total (n = 5775)	Tertile of TyG index				
		I (lowest) (n = 1445)	II (n = 1443)	III (n = 1443)	IV (highest) (n = 1444)	p
Age, years	49 ± 5	50 ± 6	49 ± 5	49 ± 5	48 ± 5	0.001
Male, n (%)	4771 (82.6)	956 (66.2)	1198 (83.0)	1292 (89.5)	1325 (91.8)	< 0.001
Systolic BP, mmHg	117 ± 14	115 ± 14	117 ± 14	118 ± 14	120 ± 14	< 0.001
Diastolic BP, mmHg	74 ± 11	72 ± 11	74 ± 11	75 ± 10	77 ± 10	< 0.001
BMI, kg/m2	24.4 ± 2.6	23.2 ± 2.6	24.1 ± 2.4	24.7 ± 2.5	25.5 ± 2.5	< 0.001
Current smoking, n (%)	2275 (39.4)	515 (35.6)	577 (40.0)	570 (39.5)	613 (42.5)	< 0.001
Total cholesterol, mg/dL	198 ± 29	188 ± 28	194 ± 28	200 ± 29	208 ± 29	< 0.001
Triglyceride, mg/dL	136 ± 79	66 ± 13	99 ± 13	139 ± 20	240 ± 82	< 0.001
HDL-C, mg/dL	54 ± 12	61 ± 13	55 ± 11	51 ± 11	46 ± 10	< 0.001
LDL-C, mg/dL	123 ± 26	114 ± 24	124 ± 25	128 ± 26	128 ± 26	< 0.001
Glucose, mg/dL	93 ± 12	87 ± 9	92 ± 10	94 ± 10	99 ± 16	< 0.001
HbA1C, %	5.5 ± 0.4	5.4 ± 0.3	5.4 ± 0.4	5.5 ± 0.4	5.6 ± 0.5	< 0.001
Creatinine, mg/dL	0.95 ± 0.16	0.91 ± 0.18	0.95 ± 0.16	0.97 ± 0.15	0.97 ± 0.15	< 0.001
TyG index	9.30 ± 0.55	8.63 ± 0.22	9.10 ± 0.10	9.46 ± 0.11	10.0 ± 0.29	< 0.001
ASCVD risk, %	3.3 ± 1.9	2.6 ± 1.9	3.2 ± 1.9	3.6 ± 1.8	4.1 ± 1.8	< 0.001
Observation time (years)	3.5 ± 1.9	3.4 ± 1.8	3.5 ± 1.9	3.5 ± 1.9	3.6 ± 1.9	0.071

**Table 2:** Impact of the TyG index (per 1 unit increase) on CAC progression based on baseline categorical CACS

	OR (95% CI)	P
Total population		
Model 1	1.73 (1.55–1.94)	< 0.001
Model 2	1.57 (1.36–1.81)	< 0.001
CACS 0		
Model 1	1.61 (1.36–1.98)	< 0.001
Model 2	1.55 (1.22–1.96)	< 0.001
CACS 1–10		
Model 1	1.62 (1.20–2.19)	0.002
Model 2	1.43 (1.03–1.98)	0.034
CACS 10–100		
Model 1	1.39 (1.10–1.75)	0.006
Model 2	1.37 (1.04–1.81)	0.025
CACS > 100		
Model 1	1.65 (1.14–2.38)	0.008
Model 2	1.41 (0.94–2.11)	0.094

Models; 1 = unadjusted; 2 = adjusted for age, sex, BMI, SBP, DBP, HDL-C, LDL-C, current smoking, and serum creatinine level

*BMI* body mass index,* SBP* systolic blood pressure,* DBP* diastolic blood pressure,* CAC* coronary artery calcification,* CACS* coronary artery calcium score,* CI* confidence interval,* OR* odds ratio,* TyG* triglyceride glucose

## Abbreviations

ASCVD: Atherosclerosis-related cardiovascular disease
BMI: Body mass index
CV: Cardiovascular
CAC: Coronary artery calcifcation
CACS: Coronary artery calcium score
CI: Confdence interval
HDL-C: Highdensity lipoprotein cholesterol
KOICA: Korea Initiatives on Coronary Artery Calcifcation
LCL-C: Low-density lipoprotein cholesterol
TyG: Triglyceride glucose
